# Supplementary material for: The highly toxic and cryptogenic clinging jellyfish Gonionemus sp. (Hydrozoa, Limnomedusae) on the Swedish west coast
Source: PeerJ. 2019 May 13;7:e6883. doi: 10.7717/peerj.6883 (PMC6521809; doi:10.7717/peerj.6883)
Supplement: Supplemental Information 1 — Each supplementary figure (1–6) show different aspects of medusae morphology. [file peerj-07-6883-s001.pdf]

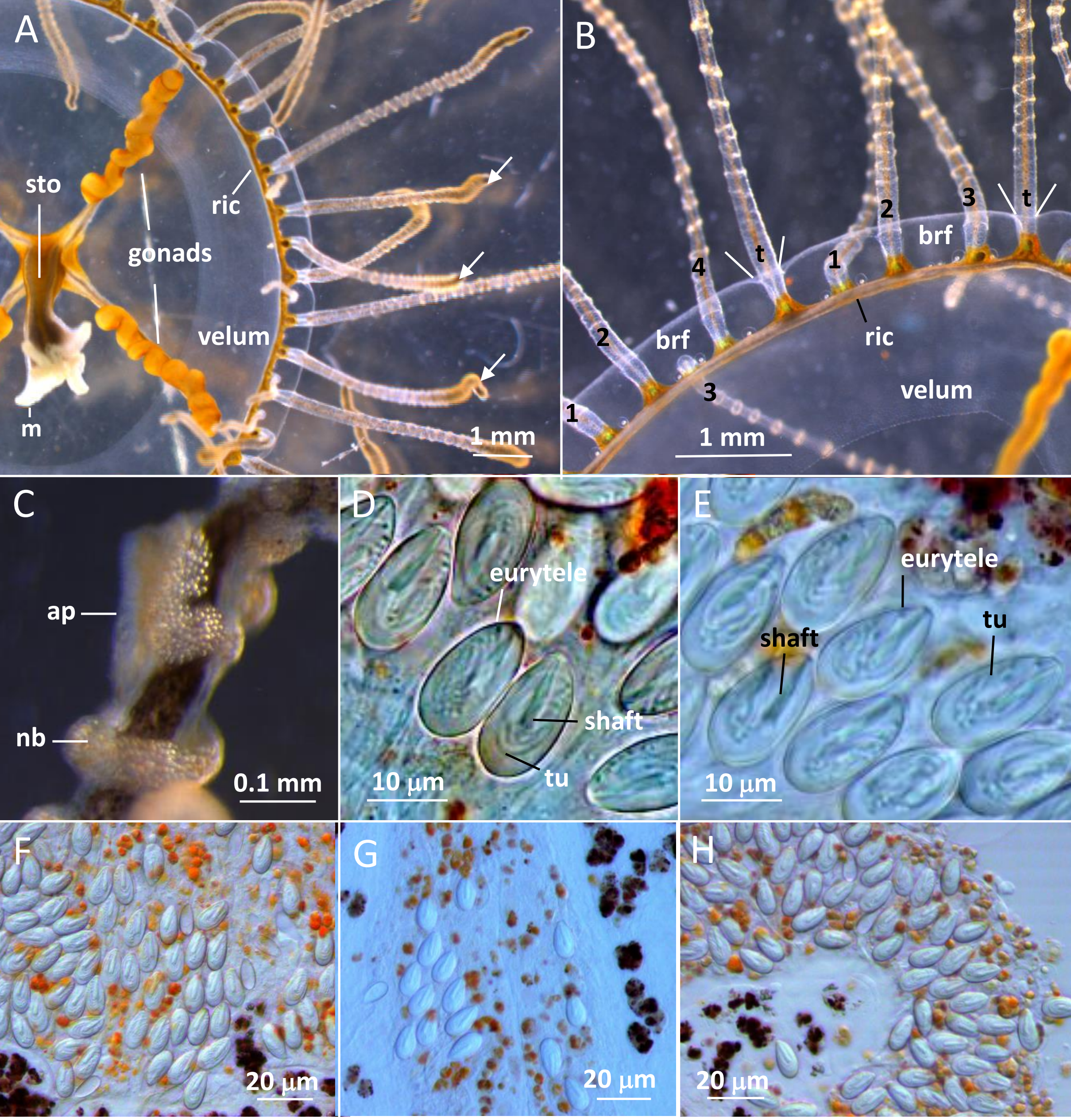

Supplementary Figure 1. (A, B) Medusa parts and tentacles in ventral view, showing immature male gonads, stomach with manubrium, velum, tentacles with nematocyst batteries, adhesive pads and ring-canal. (B) Note basal tentacles with spiral-formed nematocyst batteries and tentacle attachment sites to bell at ring-canal region. Each bell-rim flap seems to be attached to 3-5 tentacles. Tentacle (marked t, surrounded by 2 lines) is attached to 2 flaps. (C) Ring-shaped nematocyst battery and adhesive cup seen in side view. Note euryteles as small dots on adhesive cup-side. (D-H) LMs. Undischarged euryteles irregularly packed on adhesive pad-síde (D), parallel-oriented in a tentacle battery (E), packed between yellow-reddish pigments in a tentacle battery and at tentacle tip (F, H) and in a patch between batteries on pigmented tentacle mid-line (G). Abbreviations**:** *ap,* adhesive pad*; brf*, bell-rim flap; *m*, manubrium; *nb*, nematocyst battery; *ric,* ring-canal*; sh*, shaft; *sto*, stomach; ***t***, tentacle; *tu*, tubule; *1 ,2 ,3, 4,* tentacles surrounded by one bell-rim flap.

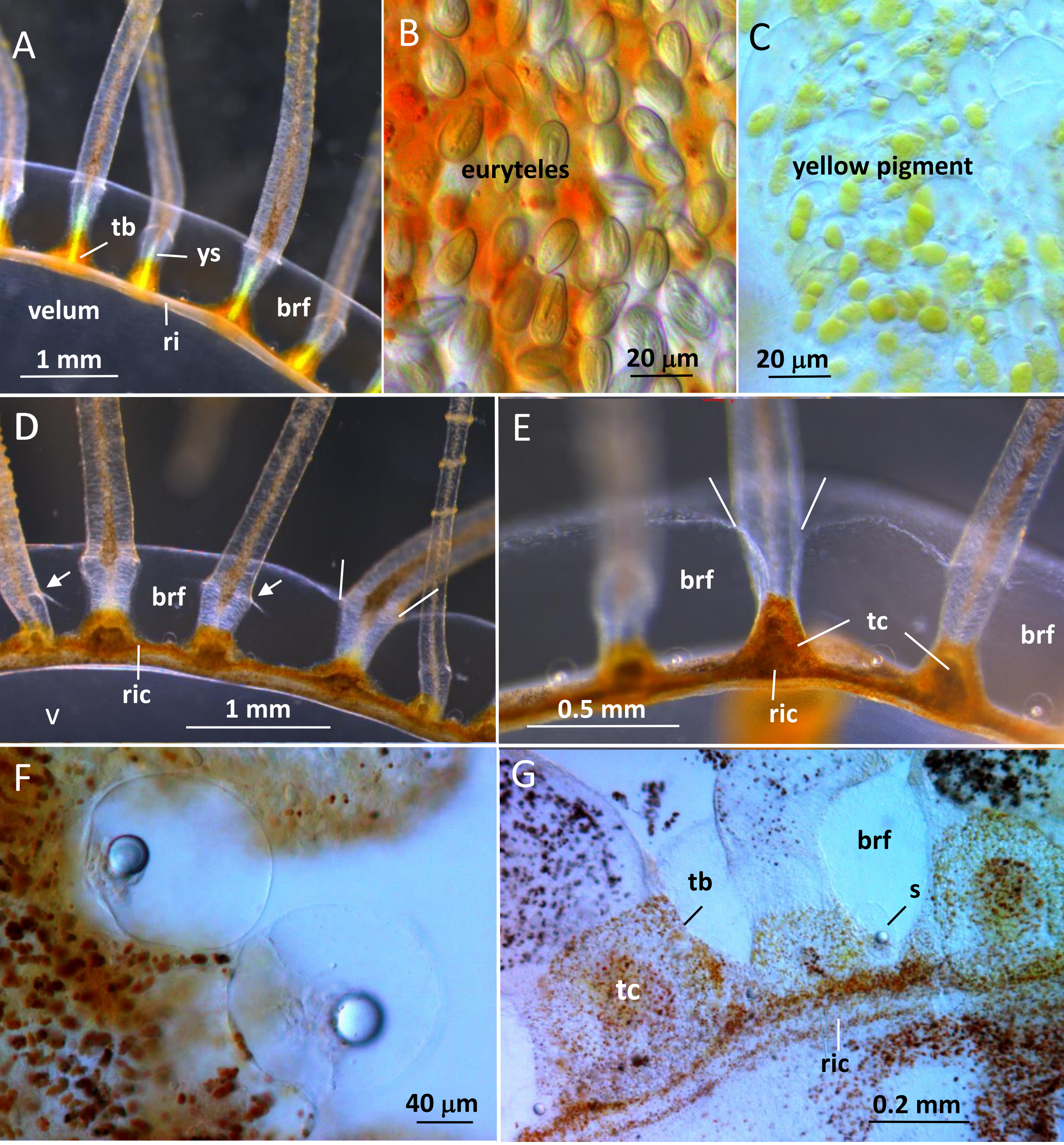

Supplementary Figure 2. Tentacles close at base seen in dorsal (A, B, C) and ventral view (D-G) showing tentacle base, yellow streak, thin young tentacle, bell-rim flap, ring-canal, tentacle-canal and statocyst. (A) Over view. (B, C) LMs (B) Euryteles from tentacle base, randomly packed among yellow-reddish pigments.(C) Yellow pigments in yellow streak. (D, E) Ventral close ups. Note attachment site of bell-rim flap to tentacle base (lines), and absence of ring formed nematocyst batteries on tentacle bases. Arrows point at possible reminiscent of attaching sites of bell-rim to tentacle in D**.** (F) Two statocysts in space between tentacles. (G) LM. Tentacle bases, showing tentacle-canal, ring-canal surrounded by pigments and statocyst. Abbreviations: *brf*, bell-rim flap; *ric,* ring-canal; *s*, statocyst; *tb,* tentacle base*; tc,* tentacle-canal*; ys,* yellow streak*; v,* velum; *white line*, attachment of bell-rim flap to tentacle.

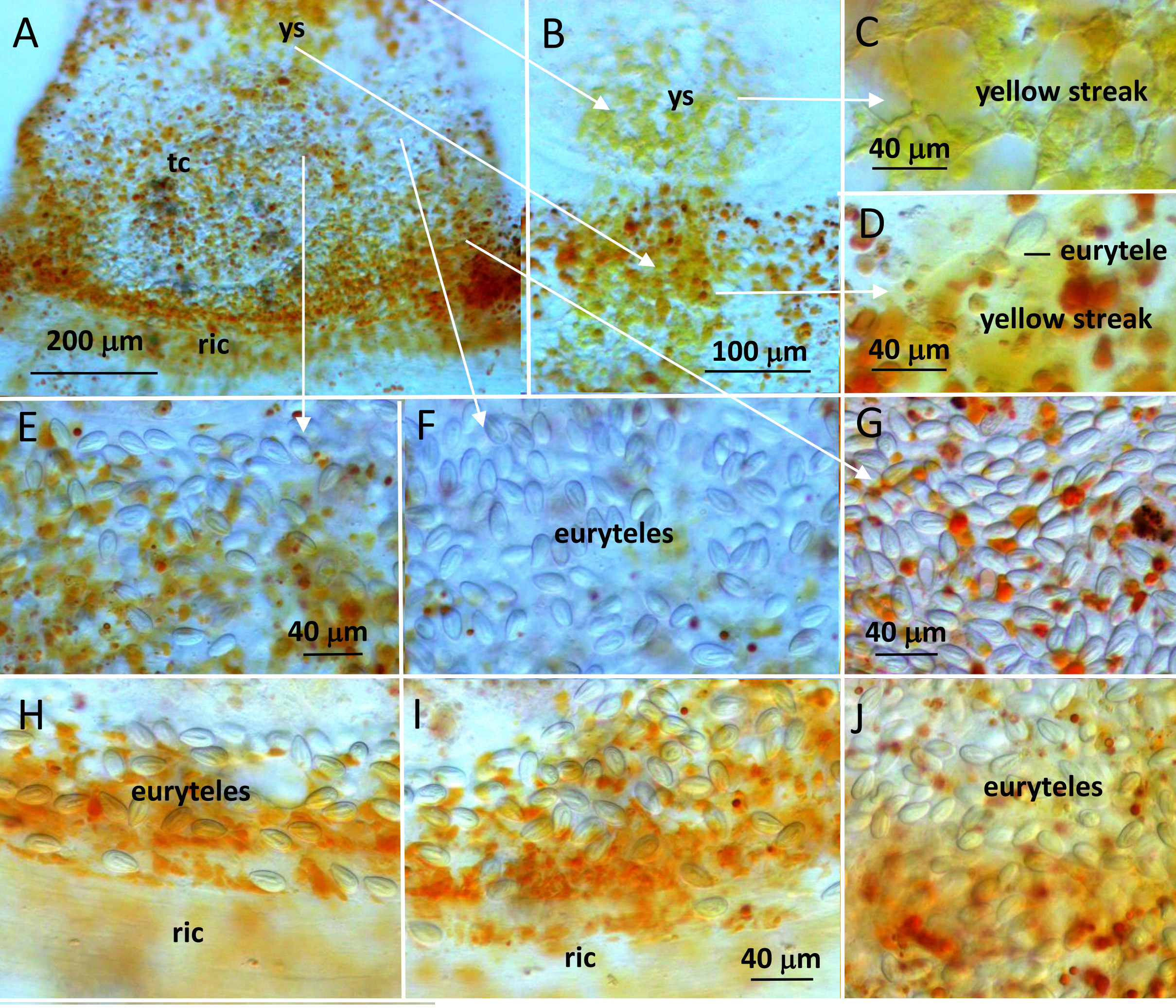

Supplementary Figure 3. LMs. (A) Tentacle base in ventral view, showing tentacle-canal (rounded structure (tc)), ring-canal, basal yellow streak and nematocyst distribution (arrow base). (B, C, D) Yellow streak with yellow pigment cells**.** (C) Close up of upper B (arrow). No nematocyst is visible. (D) Close up of lower B (arrow). One eurytele is visible. (E, F, G)**.** Close ups of A from left to right (arrows), showing euryteles randomly packed between yellow-reddish pigment cells. (H, I, J) Close ups of lower A, from left to right in the ring-canal region, showing euryteles among yellow-reddish pigment cells. (E-J) Same magnification. Abbreviations: *ric*, ring-canal; *tc*, tentacle-canal; *ys,* yellow streak.

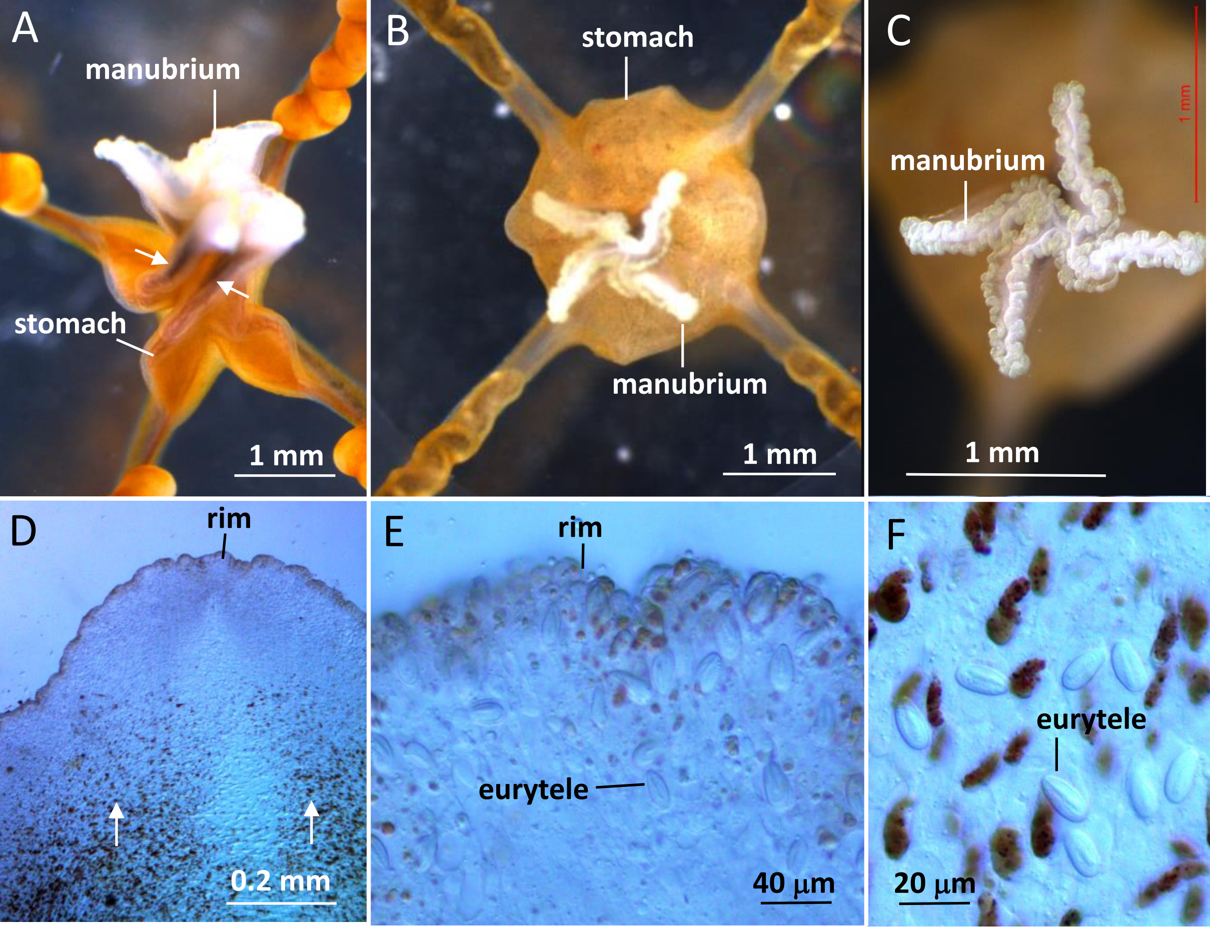

Supplementary Figure 4. Stomach, manubrium and eurytele distribution**.** (A) Empty, folded stomach attached to radial-canals. Note black pigmented streaks (arrows). (B) Filled stomach. (A-C) Manubrium mirrors the 4 radial-canals. (C) Close up of manubrium. Note undulating rim. (D-F) LMs. (D, E)**.** Parts of manubrium and stomach showing black pigmented manubrial rim, and black pigmented streaks on stomach. (E) Rim of manubrium with euryteles. (F**)** Euryteles in black pigmented streak from stomach. Abbreviations; *arrows,* point at black pigmented streaks.

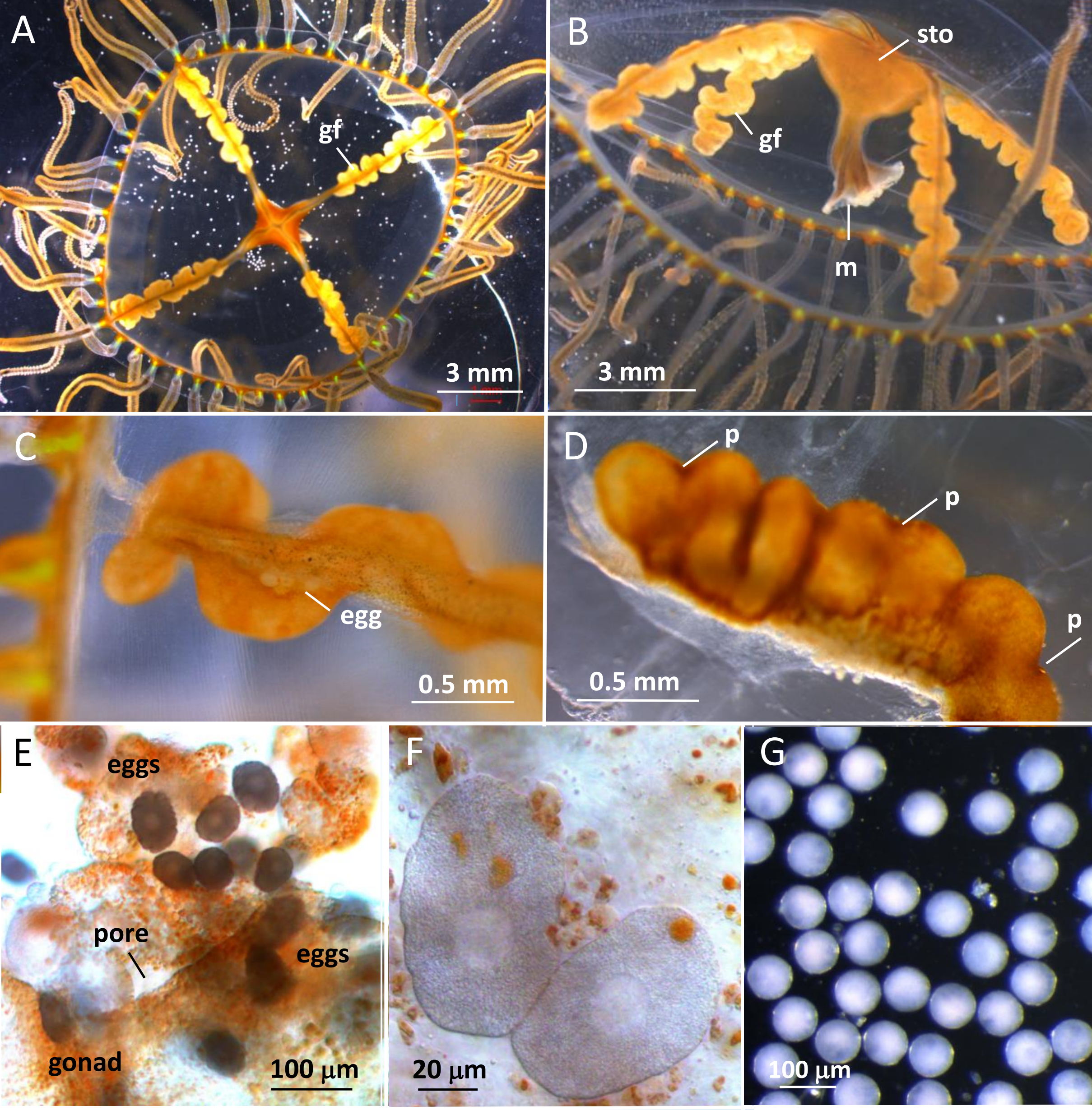

Supplementary Figure 5. (A, B) Medusa seen in dorsal (A) and lateral (B) view showing mature yellow-brown coloured female gonads attached to radial-canals. Around 8 large gonad-bulbs are visible. (A) Note ejected egg cells at bottom of the beaker. (C, D) Parts of gonad. (C) Gonad-bulbs with eggs inside. (D) Note pore, from which eggs are ejected. (E, F) LMs. (E) Eggs outside gonad with slightly undulating membrane and clearly shown nucleus compared to eggs inside gonad. (F) Two eggs with prominent nucleus and small bulbs on its surface, from inside a slightly squashed gonad. (G) Ejected immature eggs (from A). Abbreviations: *gf,* female gonad; *m,* manubrium; *p,* pore from which eggs are shed; *sto*, stomach.

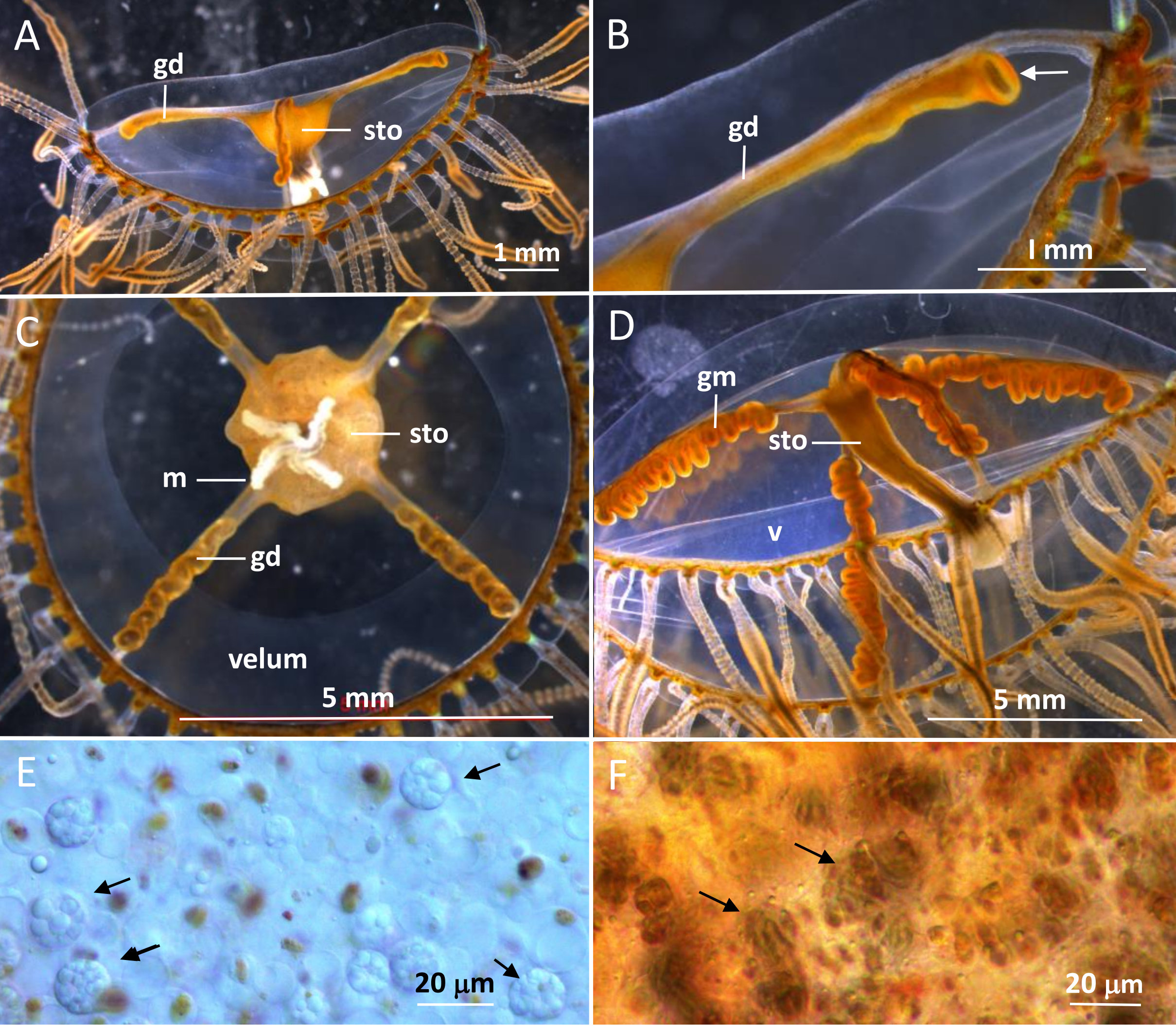

Supplementary Figure 6. Developing gonads (A-C), mature male gonad (D) and sperm development (E-F). (A) Narrow, undeveloped gonads with few poorly developed gonad-bulbs (B) Close up of A. Note pore at end of gonad (arrrow, white), from which sperms might be released. (C) Gonads, somewhat more developed compared to A, B, with several gonad-bulbs. (D) Mature male gonads, each gonad shows 12-13 small gonad-bulbs. (E, F) Developing sperm cells from inside gonad. Abbreviations: *arrows, black*, point at developing sperm cells; *arrow, whit*e, points at pore from which sperms might be released; *gd,* developing gonad; *gm*, male gonad; *m*, manubrium; *sto*, stomach; *v,* velum;
